# Complete remission of gallbladder neuroendocrine carcinoma with liver metastasis by tislelizumab plus chemotherapy: a case report

**DOI:** 10.3389/fonc.2024.1346290

**Published:** 2024-01-31

**Authors:** Huafei Li, Jiaming Qiao, Xiaoxia Kou, Cong Wu, Huiying Liu, Jinrong Qiu

**Affiliations:** ^1^ School of Life Sciences, Shanghai University, Shanghai, China; ^2^ Department of Oncology Biotherapy, The Third Affiliated Hospital of Navy Medical University, Shanghai, China; ^3^ Clinical Research Unit, The First Affiliated Hospital of Navy Medical University, Shanghai, China

**Keywords:** gallbladder neuroendocrine carcinoma, checkpoint inhibitors, tislelizumab, complete remission, case report

## Abstract

**Background:**

Gallbladder neuroendocrine carcinoma (GB-NEC) is an extremely rare cancer with a poor prognosis in the clinic. Although surgical resection remains the primary and preferred therapeutics, many patients are in a late stage and lose the opportunity for surgery. However, due to the extremely low morbidity, the specific treatment guidelines for GB-NEC have not been established.

**Case presentation:**

A 52-year-old woman was admitted to our hospital with the chief complaint of “almost 1 month after palliative surgery for metastatic gallbladder carcinoma.” According to the results of pathological findings and imaging manifestations, the patient was diagnosed with GB-NEC with a clinical stage of pT3N1M1 (IVB). The patient then received tislelizumab plus EP chemotherapy (etoposide 100 mg + cisplatin 30 mg, d1–3) every 3 weeks for 8 cycles from 12 November, 2021, followed by maintenance therapy (tislelizumab alone) every 3 weeks until now. The tumor response was evaluated as complete remission since 13 February, 2023. As of the last follow-up, the patient remains alive, with no complaints of discomfort.

**Conclusions:**

Gallbladder NEC has no specific symptoms, and the diagnosis is based on pathological and immunohistochemical results. The therapeutic course and efficacy of the case in this study indicates that the application of PD-1 inhibitor might be a feasible therapeutic option for GB-NEC. However, this potential strategy needs validation by further clinical studies in the future.

## Introduction

Neuroendocrine carcinoma (NEC) is a group of heterogeneous tumors originating from diffuse neuroendocrine cells, accounting for less than 1% of all malignancies, which commonly occurs in the gastrointestinal tract (66.0%) and respiratory tract (31.0%) (1, 2). Gallbladder neuroendocrine carcinoma (GB-NEC) is extremely rare in the clinic. From the data provided by the Surveillance, Epidemiology, and End Results (SEER) database of the National Cancer Institute (NCI), the incidence of GB-NEC was less than 0.74/100,000, accounting for 0.5% of all NECs and 2.1% of gallbladder cancers (3, 4). As previously reported, GB−NEC is more aggressive and has a poorer prognosis than gallbladder adenocarcinomas (5–7).

Currently, surgical resection remains the primary and preferred therapeutics for GB-NEC patients (8, 9). However, many patients are in a late stage and lose the opportunity for surgery. For these patients, chemotherapy is a critical treatment. According to the European Neuroendocrine Tumor Society (ENETS) 2023 guidance for digestive neuroendocrine carcinoma, platinum in combination with etoposide is recommended as the first-line treatment for patients with metastatic GB-NEC, and irinotecan with fluoropyrimidines has the best evidence as the second-line treatment. However, the regimen for adjuvant chemotherapy is not specified, although platinum/etoposide was probably used for the majority of patients (10–12). Although immunotherapy was not recommended in the guideline, the rationale for its application was based on a PD-L1 expression between 14% and 50%, a suspected high tumor mutational burden (TMB), and a broad range of multiple immune cells (13). Meanwhile, the therapeutic efficacy of immunotherapy should be thoroughly evaluated.

Herein, we describe the excellent benefits of tislelizumab, an anti-human programmed death receptor-1 (PD-1) monoclonal antibody (mAb), combined with chemotherapy in a patient pathologically diagnosed with GB-NEC with liver metastasis after palliative operation. As of the last follow-up, this patient remains alive with no complaints of discomfort, and the therapeutic response has been assessed as complete response (CR) for more than 10 months.

## Case description

**Figure 1:**
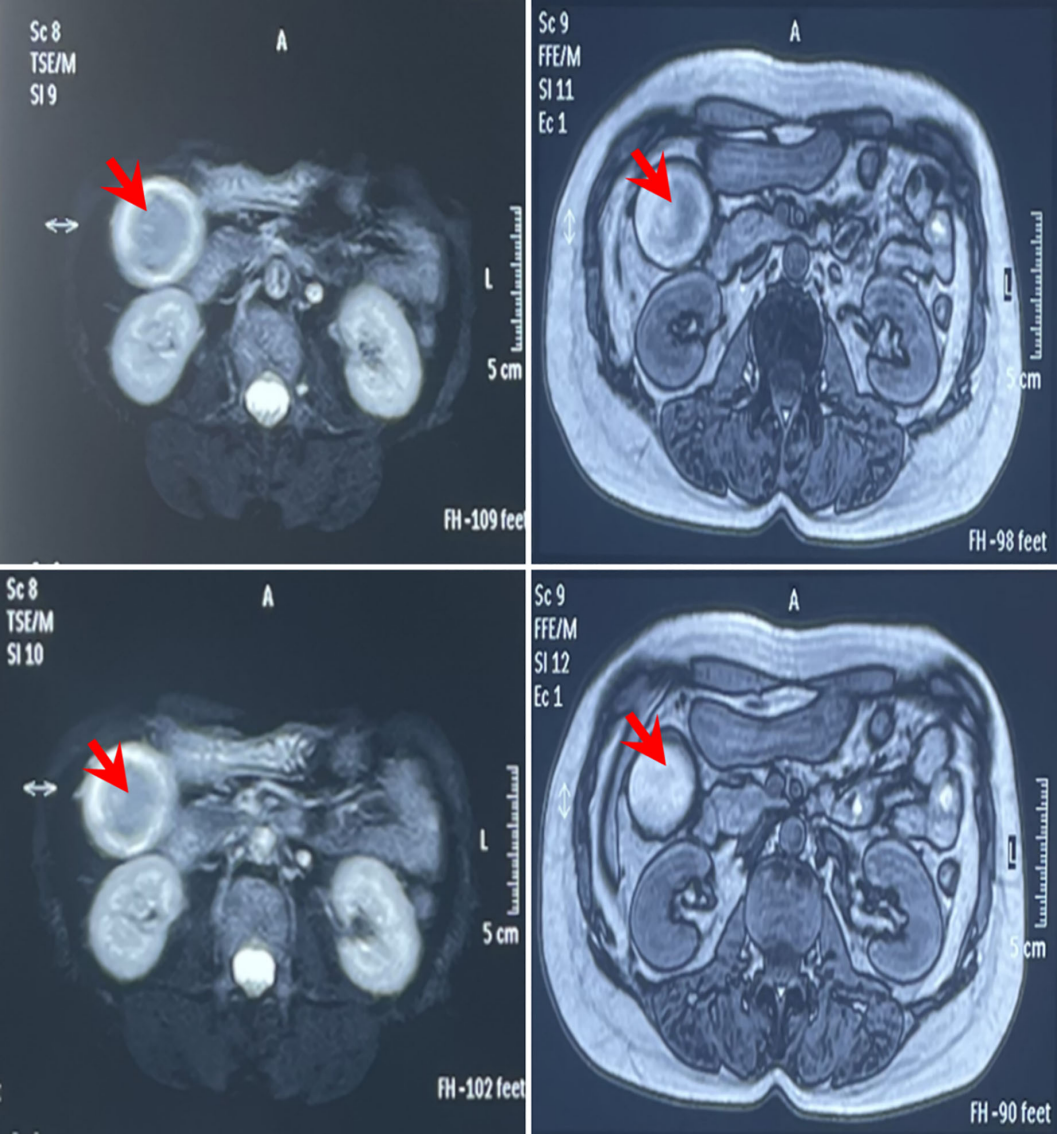
MRI imaging before operation on 9 September, 2021 of the reported case in Gaoyou People’s Hospital (Jiangsu, China). The results indicated cholecystolithiasis, cholecystitis, and cholangitis, as well as hemangioma-like changes in the left lobe of the liver (red arrow).

**Figure 2:**
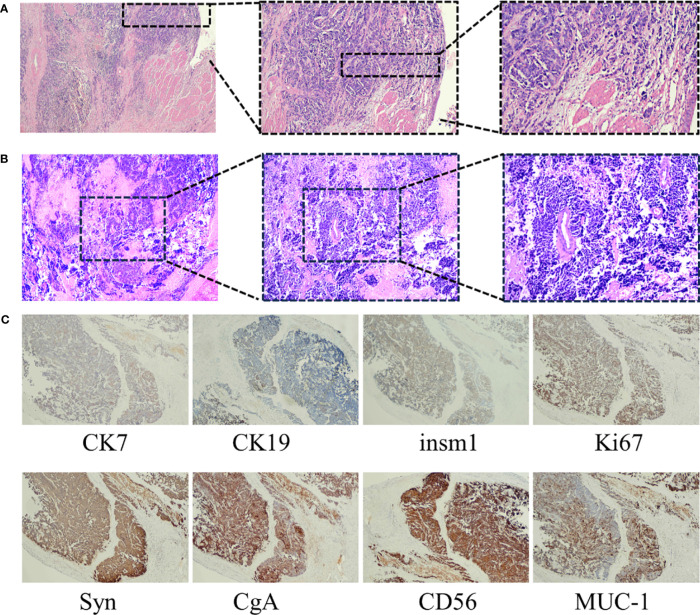
**(A)** HE staining of cancerous tissues from the gallbladder (magnification times: left panel ×40, middle panel ×100, right panel ×200). **(B)** HE staining of the liver biopsy tissues (magnification times: left panel ×40, middle panel ×100, right panel ×200). **(C)** Immunohistochemical staining showed CK7 positive, CK19 positive, INSM1 positive, Ki-67 positive, Syn, CgA positive, CD56 positive, and MUC-1-positive staining of malignant tissues (magnification times: ×40).

On 8 November, 2021, a 52-year-old woman was admitted to our hospital (The Third Affiliated Hospital of Navy Medical University, Shanghai, China) with the chief complaint of “almost 1 month after palliative surgery for metastatic gallbladder carcinoma.” On 6 October, 2021, the patient experienced unprovoked pain and discomfort in the upper abdomen, accompanied by nausea, acid reflux, and vomiting. Abdominal ultrasonography revealed cholecystolithiasis, cholecystitis, and choledocholithiasis. On October 9, the patient was admitted to Gaoyou People’s Hospital (Gaoyou, Jiangsu Province, China) for further medical care. The results of the examination after hospitalization showed the following: alanine transaminase (ALT) 161 U/L, aspartate aminotransferase (AST) 97 U/L, γ-glutamyl transferase (GGT) 246 U/L, and alkaline phosphatase (AKP) 200 U/L. No obvious abnormalities were found in the rest. Upper abdomen magnetic resonance imaging (MRI) indicated cholecystolithiasis, cholecystitis, and cholangitis, as well as hemangioma-like changes in the left lobe of the liver (**Figure 1**). Chest computed tomography (CT) and gastroscopy revealed no abnormalities. The patient had no fever, weight loss, or other complaints during the course of the disease and denied a family history of cancer. Considering the possibility of cholecystolithiasis, cholecystitis, and cholangitis, the patient underwent laparoscopic common bile duct lithotomy, laparoscopic cholecystectomy, common bile duct incision, and liver biopsy under general anesthesia on October 11. The postoperative pathology showed poorly differentiated adenocarcinoma combined with neuroendocrine carcinoma, with the cancer tissues invading the full thickness of the gallbladder wall (**Figure 2A**). In addition, a poorly differentiated neuroendocrine carcinoma was also found in the liver biopsy, which was considered to be a metastatic malignancy (**Figure 2B**).

### Diagnostic assessment

For further diagnosis and treatment, the patient was admitted to our hospital on November 8. The Eastern Cooperative Oncology Group (ECOG) score was evaluated as 1. Mild tenderness was found in the right upper quadrant without rebound tenderness. No obvious abnormalities were found for laboratory examination, except for the upregulation of neuron-specific enolase (NSE, 30.04 ng/mL). The immunohistochemistry results of the surgical specimens are as follows: insulinoma-associated protein 1 (INSM1) (+), chromogranin A (CgA) (+), synaptophysin (Syn) (+), cluster of differentiation 56 (CD56) (+), P53 (weakly positive), Ki-67 (80%+), cytokeratin 7 (CK7) (+), mucin 1 (Muc-1) (mostly +), cytokeratin 19 (CK19) (partially +), and S100P (partially +) (**Figure 2C**), supporting the diagnosis of GB-NEC. Next-generation sequencing (NGS) revealed TP53 p.S99Pfs*24 mutation, microsatellite stability (MSS), and tumor mutational burden (TMB): 3.55 Muts/Mb, and PD-L1 negative was also observed.

**Figure 3:**
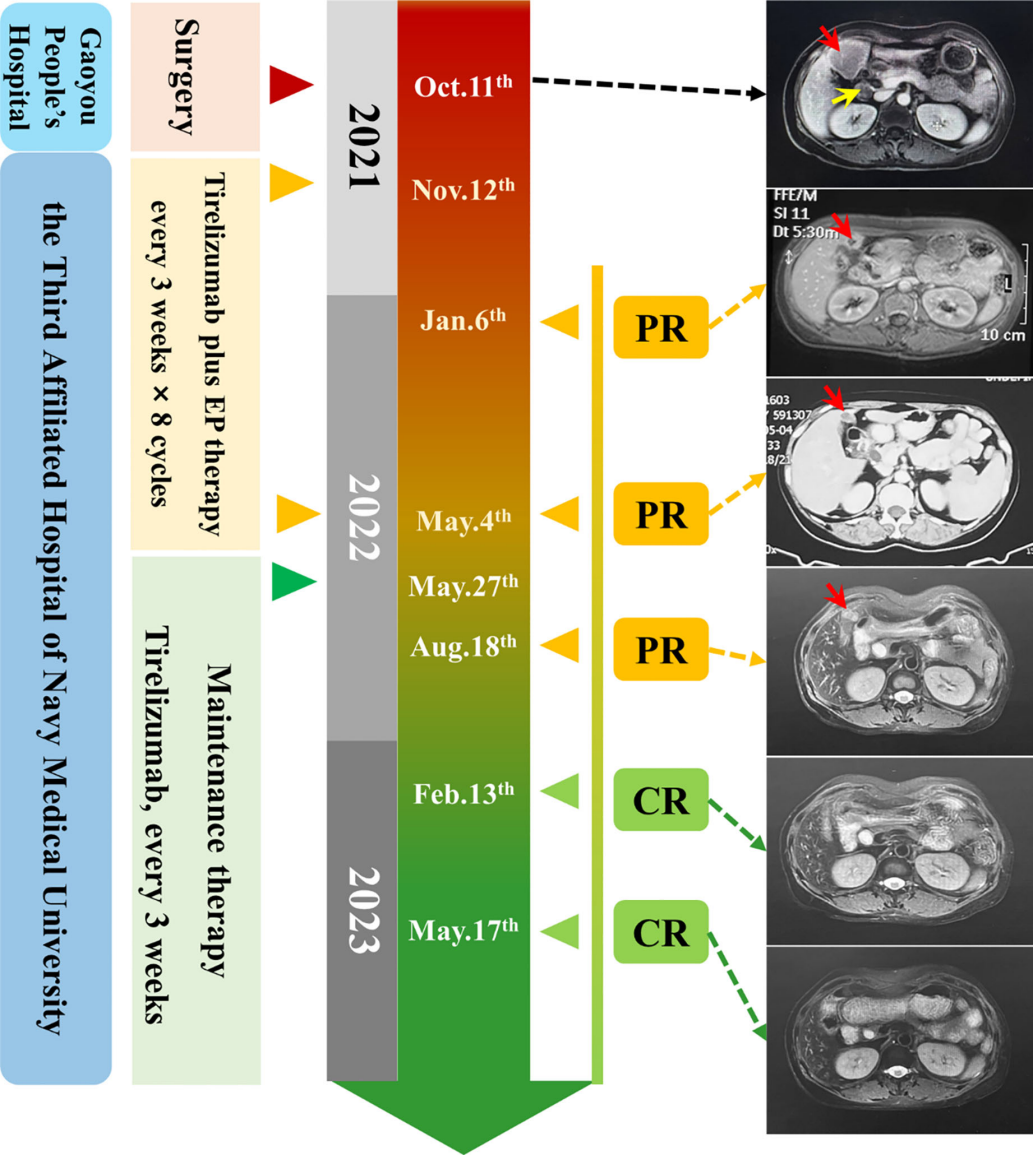
Timeline of the clinical course. Right: MRI and CT images of baseline (12 November, 2021) and after therapy (red array: metastatic cancer in the liver, yellow array: metastatic lymphoma nodes).

A single intrahepatic metastatic tumor (approximately 5.0 × 4.8 cm in size) and portal lymph node metastases (approximately 2.4 cm in diameter) were found by contrast-enhanced MRI (**Figure 3**). According to the tumor–node–metastasis (TNM) staging of the American Joint Committee on Cancer (AJCC), the tumor was classified as a clinical stage of pT3N1M1 (IVB).

### Therapeutic process

**Figure 4:**
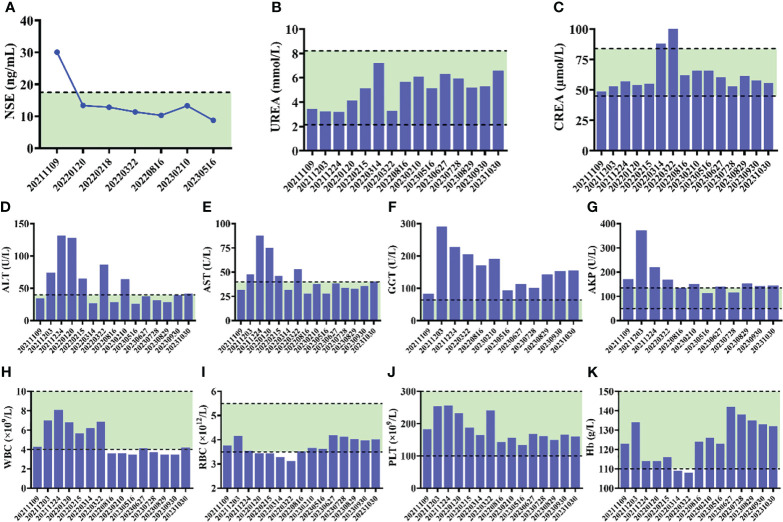
**(A)** Alteration of NSE levels of the patient during the course of therapy. **(B–G)** Serum biochemical parameters during the course of therapy. **(H–K)** The results of routine blood test during the course of therapy.

The patient received tislelizumab (200 mg, d1) plus EP chemotherapy (etoposide 100 mg + cisplatin 30 mg, d1–3) every 3 weeks for 8 cycles from 12 November, 2021. The MRI results before the third cycle of treatment (6 January, 2022) showed that the intrahepatic tumor significantly reduced to approximately 2.0 × 2.0 cm in size, and the metastatic hilar lymphoma nodes (LNs) were missed (**Figure 3**). Tumor response was evaluated as partial remission (PR). The blood NSE of the case remarkably decreased to 13.36 ng/mL before the fourth therapy and remained at normal levels since then (**Figure 4A**).

After the completion of 8 cycles of treatment (4 May, 2022), the patient was assessed as having a PR, with the size of the intrahepatic tumor further being reduced to 2.0 × 1.5 cm (**Figure 3**). The patient’s upper abdominal pain subsided, and the ECOG score was assessed as 0. During the 8 cycles of treatment, no serious adverse events (AEs) were observed (**Figure 4**).

Next, maintenance therapy using tislelizumab alone (200 mg, intravenous injection) was given every 3 weeks from 7 May, 2022 (**Figure 3**), and the therapeutic efficacy was re-evaluated as PR on 18 August, 2022. On 13 February and 17 May, 2023, the patient underwent physical examination with contrast-enhanced MRI, both results showed the disappearance of intrahepatic tumors, and the therapeutic efficacy was respectively evaluated as CR. Currently, the patient still receives regular maintenance therapy and remains alive with no complaints of discomfort at the last follow-up.

## Discussion

Because there is no distribution of neuroendocrine tissues in the gallbladder, the origin of GB-NEC remains controversial. Considering the fact that NEC was mostly accompanied by cholelithiasis, many researchers believe that GB-NEC originated from the intestinal or gastric metaplasia of gallbladder epithelium caused by chronic inflammation (9, 14). On the other hand, some researchers held the opinion that GB-NEC was derived from undifferentiated multipotent stem cells in the gallbladder tissues (9). Furthermore, other researchers insist that NECs and adenocarcinoma could undergo intertransformation, and GB-NEC possibly originated from the transformation of adenocarcinomas (9).

Clinically, GB-NEC can be divided into functional and non-functional according to whether cancerous cells can secrete peptides, causing typical symptoms such as diarrhea, flushing, edema, and wheezing. However, due to the first-pass effect on the liver, only a small fraction of cases had the secretory symptoms, and the main complaints for most patients were non-specific, such as right epigastric discomfort, poor appetite, jaundice, and weight loss, which had no value for differential diagnosis (15). Furthermore, GB-NEC lacks specific tumor biomarkers and typical imaging features by CT or MRI, making it difficult to distinguish (9). All these are important factors for the unclear preoperative diagnosis of the reported case. The confirmative diagnosis of GB-NEC requires pathological examination and immunohistochemistry, including CgA, Syn, etc., which are regarded as specific biomarkers (16).

GB-NEC is a highly malignant and aggressive disease, for which systemic metastasis is common, with the liver being the commonest site of hematogenous metastasis. Chemotherapy is critical for most patients with metastatic GB-NEC. Platinum plus etoposide chemotherapy was the most recommended first-line regimen according to the ENETS, 2023 guidance (10, 12).

In this study, when GB-NEC was pathologically confirmed after surgery, the patient received 8 cycles of EP chemotherapy plus anti-PD-1 mAb tislelizumab immunotherapy, followed by maintenance therapy using tislelizumab alone every 3 weeks. The tumor response was evaluated as PR before the third cycle of treatment and as CR after half a year of maintenance therapy. Although the NGS results indicate PD-L1 negative for the surgical specimen, the therapeutic efficacy of the tislelizumab-containing regimen remains excellent, indicating that the NGS results cannot effectively predict the efficacy of PD-1 targeted therapy.

The prognosis of GB-NEC is poor. According to previous studies, the median survival time (MST) varies from 3 months to 10 months, with 1-, 2-, and 3-year survival rates of approximately 20%, 10%, and 0% (9, 17). In this study, the treatment response of the reported case was evaluated as CR since 13 February, 2023. Although the employment of checkpoint inhibitors is rarely reported, it seems that the application of PD-1 mAb-based therapeutics following palliative operation might be a potentially effective option. However, this scheme needs validation by further clinical studies. If this information is widely available, it will accurately guide the clinical treatment of GB-NEC in the future. Moreover, although the tumor was classified as clinical stage pT3N1M1 (IVB) in this case, according to the perioperative examination results, the intrahepatic tumor and lymph nodes were the metastatic sites only found. Thus, the limited extent of metastasis might also be the reason for the excellent therapeutic efficacy.

## Data availability statement

The original contributions presented in the study are included in the article/supplementary material. Further inquiries can be directed to the corresponding authors.

## Ethics statement

Written informed consent was obtained from the individual(s) for the publication of any potentially identifiable images or data included in this article.

## Author contributions

HFL: Conceptualization, Data curation, Formal analysis, Funding acquisition, Writing – original draft, Writing – review & editing. JMQ: Data curation, Formal analysis, Investigation, Writing – original draft, Writing – review & editing. XK: Writing – original draft. CW: Data curation, Formal analysis, Investigation, Writing – review & editing. HYL: Investigation, Methodology, Project administration, Resources, Supervision, Visualization, Writing – review & editing. JRQ: Conceptualization, Data curation, Formal analysis, Investigation, Project administration, Resources, Visualization, Writing – review & editing.
